# A Major QTL for Resistance to *Vibrio anguillarum* in Rainbow Trout

**DOI:** 10.3389/fgene.2020.607558

**Published:** 2020-12-29

**Authors:** Asma M. Karami, Jørgen Ødegård, Moonika H. Marana, Shaozhi Zuo, Rzgar Jaafar, Heidi Mathiessen, Louise von Gersdorff Jørgensen, Per W. Kania, Inger Dalsgaard, Torben Nielsen, Kurt Buchmann

**Affiliations:** ^1^Laboratory of Aquatic Pathobiology, Department of Veterinary and Animal Sciences, Faculty of Health and Medical Sciences, University of Copenhagen, Copenhagen, Denmark; ^2^AquaGen, Trondheim, Norway; ^3^Institute of Aquatic Resources, Technical University of Denmark, Kongens Lyngby, Denmark; ^4^Aquasearch Ova ApS, Jelling, Denmark

**Keywords:** QTL, rainbow trout, *Vibrio*, immunity, gene expression

## Abstract

Genetic selection of disease resistant fish is a major strategy to improve health, welfare and sustainability in aquaculture. Mapping of single nucleotide polymorphisms (SNP) in the fish genome may be a fruitful tool to define relevant quantitative trait loci (QTL) and we here show its use for characterization of *Vibrio anguillarum* resistant rainbow trout (*Oncorhynchus mykiss*). Fingerlings were exposed to the pathogen *V. anguillarum* serotype O1 in a solution of 1.5 × 10^7^ cfu/ml and observed for 14 days. Disease signs appeared 3 days post exposure (dpe) whereafter mortality progressed exponentially until 6 dpe reaching a total mortality of 55% within 11 days. DNA was sampled from all fish – including survivors – and analyzed on a 57 k Affymetrix SNP platform whereby it was shown that disease resistance was associated with a major QTL on chromosome 21 (Omy 21). Gene expression analyses showed that diseased fish activated genes associated with innate and adaptive immune responses. The possible genes associated with resistance are discussed.

## Introduction

The bacterial pathogen *Vibrio anguillarum* is the causative agent of classical vibriosis. It is likely one of the earliest recognized bacterial fish diseases as the causative pathogen was isolated and described from *Anguilla anguilla* by Bergman (1909). However, infections target a large number of wild and aquacultured fish species varying from marine to freshwater species distributed in temperate and subtropical regions (Toranzo and Barja, 1990). The disease is associated with hemorrhagic septicemia, anorexia, hemorrhages (skin and fin bases), abscesses, ulcerations and high mortality (Actis et al., 1999). *V. anguillarum* is a motile Gram-negative curved rod with polar flagella and its virulence factors comprise chemotaxis, proteases, hemolysins, LPS structure and siderophore production. A serotype system is applied to differentiate different serovars within the species and it has been shown that serotype O1 mainly infects salmonids whereas serotype O2 preferentially infects cod (Larsen et al., 1994). Almost all salmonid species, including rainbow trout (*Oncorhynchus mykiss*), are susceptible to the bacterium which utilizes different entrance portals (gills, skin, mouth, anus, and intestine) when first colonizing the host (Larsen et al., 1988). Mariculture of salmonids in general and rainbow trout farming in particular have for decades suffered from vibriosis caused by *V. anguillarum* (Pedersen et al., 2008) and various strategies based on administration of antibiotics and immunoprophylaxis have been applied for control (Holten-Andersen et al., 2012). Rainbow trout is able to mount a protective immune response against the infection (Harrell et al., 1975; Viele et al., 1980) and intraperitoneal injection of simple bacterins, based on formalin-killed strains of *V. anguillarum*, are known to reduce morbidity and mortality (Thornburn and Jansson, 1988; Håstein et al., 2005; Brudeseth et al., 2013; Marana et al., 2019). These studies have shown that mortality may exceed 50% in the unprotected population. However, despite the implemented immunoprophylactic measures, outbreaks of vibriosis at high sea-temperatures in summer periods still occur and call for antibiotic treatment (Pedersen et al., 2008). In addition, even successful vaccination programs are costly, as the majority of maricultured fish are vaccinated (Danmap, 2019) and expenses associated with the vaccine purchase and labor expenses reach approximately 0.1 euro per fish. Far higher expenses are associated with production losses (several percent daily growth loss) when starving fish before and after vaccination. It is therefore relevant to apply additional protective measures for optimization of fish health and one sustainable strategy is to apply selective breeding of naturally resistant fish strains for aquaculture. Classical selection programs, utilizing exposed but surviving fish as breeders for the coming generations, demand several years to obtain results at farm level (Gjedrem and Baranski, 2009). Other approaches are based on the use of markers (Fraslin et al., 2020) identifying individual fish with traits for disease resistance. Quantitative trait loci (QTL) associated with disease resistance in rainbow trout have been identified by application of molecular markers (e.g., microsatellites or SNPs) that are associated with genes conferring resistance toward diseases such as infectious pancreatic necrosis (IPN) (Ozaki et al., 2007), viral hemorrhagic septicemia (VHS) (Verrier et al., 2013) and bacterial cold water disease (BCWD) (Wiens et al., 2013; Vallejo et al., 2014; Fraslin et al., 2018). A series of studies employing linkage analysis (LA) and Genome-Wide Association Studies (GWAS), led to the production of a 57 K array, detecting single nucleotide polymorphisms (SNP) and their location on the 29 chromosomes in rainbow trout (Palti et al., 2015; Pearse et al., 2019). This mid-density genotyping array has proven to be of great value for investigating the genetic basis of phenotypic variation and host-pathogen interactions. The applicability of the 57 K SNP array (Affymetrix Axiom^®^) was confirmed for detection of QTL associated with rainbow trout resistance against infectious hematopoietic necrosis virus (IHNV) (Vallejo et al., 2019), columnaris disease (Silva et al., 2019), BCWD (Vallejo et al., 2017; Liu et al., 2018), enteric red mouth disease (ERM) (Zuo et al., 2020) and white spot disease (WSD) (Jaafar et al., 2020). Correspondingly, an array developed for Atlantic salmon (*Salmo salar*) was applied to detect QTL associated with resistance in this host species against salmonid alphavirus (SAV) (Hillestad et al., 2020).

Genetic breeding for disease resistance has been successfully implemented in the industry. Based on previous studies on genes associated with IPNV resistance in Atlantic salmon (Houston et al., 2008; Moen et al., 2009) new breeding programs have resulted in 75% decrease in IPN outbreaks in the salmon farming industry (Moen et al., 2015; Norris, 2017). Recent investigations have also identified QTL associated with *V. anguillarum* resistance in non-salmonid species including Japanese flounder (Wang et al., 2014; Shao et al., 2015), turbot (Zhang et al., 2019) and tonguefish (Tang et al., 2016). QTL associated to resistance against this disease in rainbow trout have remained unexplored but in the present study we describe a major QTL connected to resistance. The current study was performed in order to elucidate the natural resistance in rainbow trout against vibriosis caused by *V. anguillarum* (serotype O1). We exposed 800 rainbow trout to the pathogen and followed the mortality over 14 days. DNA samples from all fish were recovered and analyzed using the 57K array, which allowed us to identify a major QTL for vibriosis resistance. In addition, during the course of infection we sampled fish for gene expression analysis in order to identify immune gene expression profiles in the different groups with different susceptibility to infection and point to possible resistance mechanisms in rainbow trout.

## Materials and Methods

### Fish

Certified virus free rainbow trout eyed eggs, originating from an out-bred population based on 72 families (Aquasearch ova ApS, Jutland, Denmark), hatched at 7°C during a 14 day period (March 2019) at a certified pathogen-free hatchery [Aqua Baltic, Nexø, Denmark (Xueqin et al., 2012)]. The breeding set-up for these families applied eggs from one female for two families and milt from one male to four families. Following bacterial exposure, sampling and genotyping, a total of 669 fish were used for analysis and it is estimated that 9–10 fish from each of the 72 families are represented in the material. After rearing to the juvenile stage at 12°C (reaching a body weight of 12 g (range 11–13 g), 2880 degree-days post-hatch) the fish were transported (3 h transfer time at oxygenated and cooled conditions) to the infection facility at the University of Copenhagen (Frederiksberg, Denmark). Before the challenge, bacterial and parasitological examination was performed, confirming absence of pathogens (Dalsgaard and Madsen, 2000; Buchmann, 2007). A total number of 800 fish used for bacterial infection and 200 fish used as non-infected controls were kept and acclimatized in separate plastic tanks (separate rooms) (INTEX^®^, 2.60 m × 1.60 m × 0.65 m, total volume 2.7 m^3^) each containing 800 L of municipal water (certified chlorine free water, Frederiksberg commune with 14 days pre-aeration before use). Temperature was kept at 18°C (temperature favoring vibriosis outbreaks at farm level) (Haenen et al., 2014; Marana et al., 2019). Water was recycled (20 L/min) by internal biofilters (EHEIM, Germany) applying 30% water replenishment daily. Water quality was monitored and kept constant at pH 7.6, nitrite <0.01 mg/l, nitrate <50 mg/l (TetraTest 6in1, cat.no. T704154, Eldorado A/S, Denmark) and ammonia <0.5 mg/l (API Ammonia Test Strips, VioVet Ltd., United Kingdom). During the 14 days acclimation and 14 days experimental infection period, fish received commercial pelleted feed (1.5% biomass per day, INICIO 917, BioMar A/S, Denmark).

### Ethics Statement

Infection procedures were performed under the license 2019-15-0201-01614 obtained from the Experimental Animal Inspectorate, Committee for Experimental Animals, Ministry of Environment and Food, Denmark. Ethical guidelines of the University of Copenhagen were followed securing that fish were monitored every second hour around the clock. Death is not acceptable as humane endpoint (EU directive 2010) and therefore fish showing clinical signs (loss of equilibrium, irregular swimming, bleedings, discoloration) were euthanized with overdose (300 mg/L) of ethyl-3-aminobenzoate methanesulfonate (MS222) (cat.no. A5040, Sigma-Aldrich, Denmark) and recorded as mortality.

### Challenge Procedure

Fish were exposed to the pathogen for 2 h at 18°C in a partly drained fish tank containing 450 L of *Vibrio anguillarum* culture (serotype O1, isolate 130829-1/3, concentration 1.5 × 10^7^ cfu/ml). Three L broth (Veal Infusion Broth, Difco 0344176, United States) of a 48 h laboratory culture (1.2 × 10^9^ cfu/ml) was added into the tank containing 450 L tap water. Following the challenge procedure, municipal water (chlorine free and aerated as specified above) was added to the tank to reach a total volume of 800 L. Fish were kept as described above for acclimatization and observed for occurrence of any disease signs for 14 days. Non-exposed control fish were kept under the same conditions but in a separate non-infected room.

### Sampling

Mortality was recorded from the exposure time point to 14 days post exposure (dpe). Mortality stopped after 11 dpe but fish were kept for another 3 days to confirm absence of disease signs in survivors.

#### Sampling for Genotyping

Samples were taken from all fish throughout the course of infection, first by sampling the susceptible fish (recorded as mortality) and in the end all the fish surviving the challenge (resistant group). Samples for DNA-typing (a circular tissue piece, diameter 2.75 mm, of the tail fin) were taken using a punching scissors (agnthos.se, Sweden) and transferred to lysis buffer (Vaxxinova.no) for subsequent DNA extraction, purification and genotyping. Genotyping was done on a 57 k SNP chip, Affymetrix^®^ (San Diego, United States) developed for rainbow trout (Palti et al., 2015) and performed according to the Axiom^®^2.0 Assay-Automated-Workflow-User-Guide Affymetrix_1 (2020). Based on the raw data provided by the Axiom machinery, genotypes were called using the Affymetrix-Power-Tools-software. The analysis and interpretation of the raw data was done according to the Best Practices Workflow (Affymetrix_2, 2020).

#### Sampling for Gene Expression Analysis

Gills, liver and spleen were sampled from fish on day zero (before bacterial exposure), 3 dpe when disease signs (equilibrium disturbance, lethargy) developed, including fish with clinical signs CS (15 fish) and without clinical signs NCS (15 fish) and on 11 dpe (15 survivors). For each time point (0, 3, and 11 dpe), 15 control fish (not exposed to the bacterium) were taken in parallel. Samples were fixed in RNAlater (cat.no R0901, Sigma-Aldrich, Denmark), placed at 4°C for 24 h and subsequently stored at −20°C until further processing.

### Quantitative RT-qPCR (qPCR)

Primers and Taq-Man probes applied are listed in Supplementary Table 1 (previously designed and described by Purcell et al., 2004; Ingerslev et al., 2006; Raida and Buchmann, 2007, 2008, 2009; Sugiura et al., 2007; Olsen et al., 2011; Chettri et al., 2012, 2014; Holten-Andersen et al., 2012; Skov et al., 2012, 2018; Jaafar et al., 2015; Marana et al., 2017) and the gene expression analyses were performed as described by Zuo et al. (2020). In brief, samples from fish (gills, liver, and spleen) were homogenized (Tissue-lyser II, Qiagen, Denmark) using a homogenization buffer with 2-mercaptoethanol (Sigma-Aldrich). RNA was extracted using the GenElute^TM^ mammalian RNA kit (RTN350, Sigma-Aldrich, Denmark). Liver samples were pre-treated by Proteinase K (cat.no.P4850, Sigma-Aldrich, Denmark). DNase I (AMPD1, Sigma-Aldrich, Denmark) treatment was applied to remove genomic DNA. The concentration of RNA was determined in a NanoDrop 2000 spectrophotometer (Saveen & Werner, Denmark). Quality of RNA was evaluated by agarose (cat.no. 16500100, Thermo Fisher Scientific, Denmark) gel electrophoresis. RNA was kept at −80°C until cDNA synthesis in T100 thermocycler, Biorad, Denmark, using Oligo d(T)16 primer and TaqMan^®^ Reverse Transcription Reagents (cat.no. N8080234, Thermo Fischer Scientific, Denmark). Finally, cDNA was stored at −20°C until further use. Quantitative PCR assays were performed using an AriaMx Real-Time PCR machine (cat.no.G8830A-04R-010, AH diagnostics AS, Denmark). The cycling conditions were one cycle of predenaturation at 95°C for 3 min. This was followed by 40 cycles of denaturation at 94°C for 5 s with a combined annealing/elongation process at 60°C for 15 s with endpoint measurement. Primers and Taq-Man probes targeting immune-relevant rainbow trout genes were synthesized at TAG Copenhagen AS, Denmark. Reaction volumes were 12.5 μl [2.5 μl cDNA, 6.25 μl Brilliant III Ultra-Fast QPCR Master Mix (600881, AH Diagnostics AS, Denmark), 1.0 μl primer-probe mixture (forward primer, 10 μM and reverse primer, 10 μM), Taq-Man probe (5 μM), and 2.75 μl RNase-free water]. Wells with sample without enzyme (reverse transcriptase minus) and negative controls (water instead of sample) were used for every plate setup. NormFinder (Andersen et al., 2004) was applied to evaluate all combinations of three reference genes for use as endogenous control. These were elongation factor (ELF) 1-α, β-actin and acidic ribosomal phosphoprotein P0 (ARP). The average of the three genes was chosen and stability values were 0.002, 0.005, and 0.002 for liver, spleen and gills, respectively. In order to quantify the bacterial load in different organs at the different time points, we measured the gene expression level of the bacterial gene *recA* (GenBank ass.no. LC370212), which was described as a household gene in *V. anguillarum* (GenBank ass.no. LC370212) by Crisafi et al. (2014). We designed the probe for the qPCR assay according to Zuo et al. (2020) with minor modifications. For efficiency determination (estimated 102%), a dilution series of samples with high expression of *V. anguillarum* recA was used. Specificity, was tested by SYBER green and melting curve analysis confirmed by electrophoresis, product length 248 bp. The bacterial transcript level was estimated as 2^–Δ^ ^Cq^. It should be noted that dead or inactive bacteria may not be detected by this method.

### Data Analysis

#### Survival

Kaplan-Meier survival analysis (GraphPad Prism version 48.3.1, Bethesda, ML, United States) was used for estimation of cumulative mortality rates.

#### Gene Expression

All q-PCR assays were developed and tested in dilution series with five replicates and exhibited efficiencies of 100 ± 5% (presence of inhibitory molecules in the sample may raise the efficiency above 100%). This allowed the use of the simplified 2^–Δ^ ^Δ^ ^Cq^ method for analyzing data (Livak and Schmittgen, 2001). For normalization and as internal calibrator, the average of the three genes (ARP, ELF1α, and β-actin) was chosen by using NormFinder (Andersen et al., 2004). One-way ANOVA with Tukey’s test was applied on gene expression results from day 3 (multiple comparison between CS, NCS and control groups) and Student’s *t*-test was used to compare results on day 11. We considered differences to be significant when regulations were at least two-fold and *p* < 0.01. For four of the genes investigated in the non-exposed timepoint control groups the transcriptions were so low that less than three fish showed Cq values. This excluded the use of a *t*-test and we therefore applied a qualitative assessment (presence/absence of Cq values) and analyzed data with the non-parametric Mann–Whitney test using a probability level of 5%.

#### Genetic Analysis

For the genetic analyses, only fish of high genotype quality were included in the analysis, resulting in a dataset of 669 individuals (representing 9–10 fish from each family), of which 295 survived and 374 were recorded as mortalities. SNPs were restricted to those classified as “PolyHighResolution” by the Thermo Fisher Array Power Tools (APT) software. MAF > 0.01 was required. This means that the SNP genotypes are considered high quality and polymorphic (all three possible genotype clusters are present) resulting in 35,200 SNP loci being included in the analysis. By applying all the high-quality SNPs on the SNP chip, a genomic relationship matrix (GRM) was computed by the GCTA software, using the default algorithm (Yang et al., 2010). This was used to account for polygenic effects (including family background effects). Fish from the same full-sib family necessarily shared a large fraction of alleles, implying that their relationship was high in the GRM (as would also be the case for a pedigree-based relationship matrix). In the GWAS, we utilized a leave one chromosome out approach. This means that the GRM was set up using all SNPs, except those located on the same chromosome being currently tested (so that the GRM did not capture the effect of potential QTL on that chromosome, but general polygenic effects were captured). Using a linear model, heritability was estimated on the observed scale. The h2 was transformed to the underlying scale (Table 1) using the built-in options of GCTA based on the theory and formulas found in Lee et al. (2011). Statistical analysis was performed using the software package GCTA (Yang et al., 2011). The data set was initially analyzed with a simple genomic animal model using REML:

**TABLE 1:** Estimated variance components from REML analysis of rainbow trout resistance to *V. anguillarum*.

**Factor**	**Estimate**	**SE**
Additive genetic variance	0.057312	0.019821
Residual variance	0.200958	0.015421
Phenotypic variance	0.258270	0.016627
Heritability (observed scale)	0.221907	0.068252
Heritability (underlying scale)	0.349595	0.107526

y=1⁢μ+g+e.

where y is a vector of phenotypes (0 = dead, 1 = survived), *μ* is the overall mean, $g∼N\,\left(0,G\,\sigma_{g}^{2}\right)$ is a vector of additive genetic (polygenic) effects, G is the genomic relationship matrix (Yang et al., 2010), computed from all high-quality markers, $\sigma_{g}^{2}$ is the polygenic variance, $e∼N\,\left(0,I\,\sigma_{e}^{2}\right)$ is a vector of random residuals and $\sigma_{e}^{2}$ is the residual variance.

Secondly, leave one chromosome out genome-wide association study (LOCO-GWAS) was performed using the same model as above but extended with individual SNP effects. Here, polygenic effects on all other chromosomes except the one being currently tested are accounted for. The SNP effects were solutions from the linear mixed model LOCO-GWAS model. This model includes a fixed overall mean, a fixed regression using the SNP being tested as a covariate (the estimated SNP effect is the solution for this regression coefficient) and a random polygenic animal effect (with GRM estimated from all chromosomes except the one being currently tested). Genomic inflation factor (over the entire genome) was 1.068081 (near the expected 1.0). QQ plot (Supplementary Figure 2) demonstrate that the model has sufficiently dealt with possible population stratification.

In order to elucidate a possible association between the immune related genes targeted in the qPCR study and the SNP results, we localized the genes in the rainbow trout genome (Pearse et al., 2019). Probe sequences were mapped against the reference genome (Omyk_1.0: GCF_002163495.1) using Burrows-Wheeler Alignment tool (BWA) (Li and Durbin, 2009). SNPs were identified from the CIGAR string of mapped probes using the HTSeq-python package (Anders et al., 2015).

## Results

### Mortality

**FIGURE 1:**
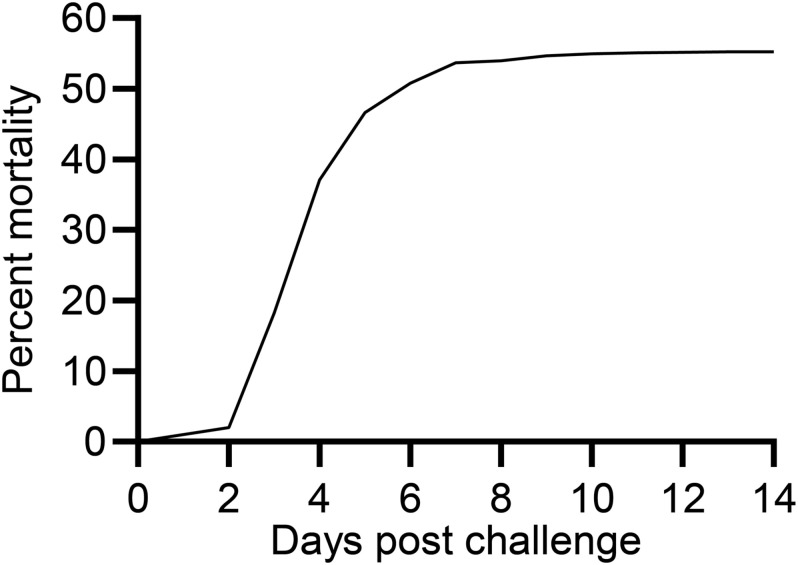
Mortality curve for rainbow trout exposed to *V. anguillarum*.

The first moribund fish was recorded at 3 dpe whereafter the mortality increased exponentially, continued at 4 dpe, decelerated at 5 and 6 dpe and ended at 11 dpe with a total mortality of 55% (Figure 1).

### Genetic Analysis

**FIGURE 2:**
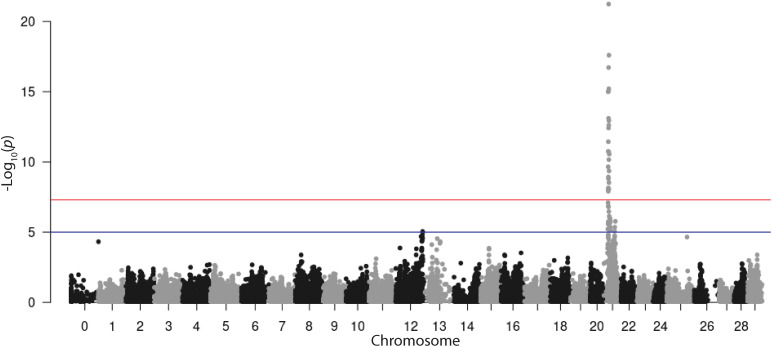
Manhattan plot from an LMM-LOCO GWAS of time to death in rainbow trout after exposure to *V. anguillarum*.

**FIGURE 3:**
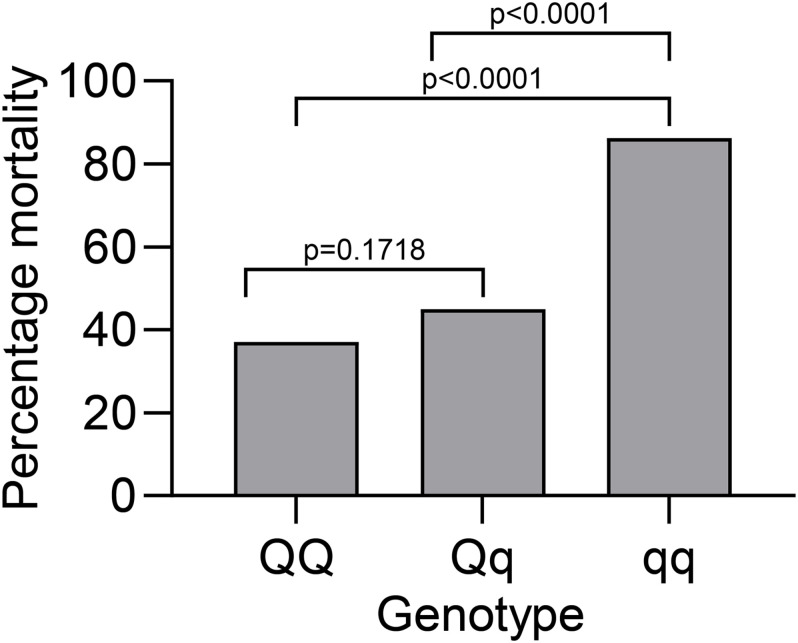
Average mortality of exposed rainbow trout to *V. anguillarum* with different genotypes for the top SNP at chromosome 21 (AX-89945921).

**FIGURE 4:**
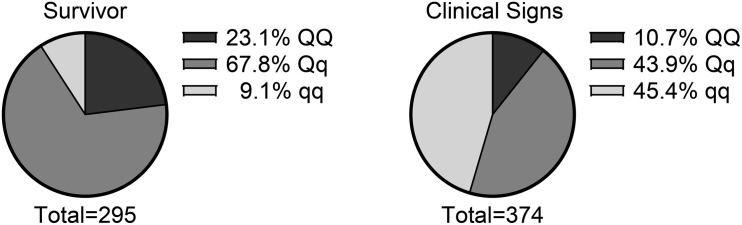
Distribution of three different genotypes (based on the top SNP at chromosome 21, AX-89945921) in moribund and surviving rainbow trout exposed to *V. anguillarum*.

The REML analysis (Table 1) presents the estimated variance for additive genetic effects (0.05 ± 0.01), heritability of vibriosis resistance on both observed (22%) and liability (35%) scales. Genetic markers on chromosome 21 showed significant association with vibriosis resistance and the high genotype quality on that chromosome clearly indicating the presence of a QTL (Table 2 and Figure 2). The SNP (AX-89945921) had a high minor allele frequency (43%, Table 1), indicating a potential for genetic improvement. The average observed mortality for fish having different genotypes, based on the most significant SNP (AX-89945921) explaining 77% of the genetic variance, was calculated. Furthermore, inclusion of the most significant SNP as a cofactor in the statistical analysis reduced the additive genetic variance by 43%. This means that a substantial fraction of genetic variance is explained by this single top SNP. Specifically addressing this SNP the favorable homozygote (QQ) has an overall mortality of 37%, while the opposite homozygote (qq) has a mortality of 87% and the heterozygote (Qq) has a similar resistance (45% mortality) as the favorable homozygote (QQ) (Figure 3). Estimated fractions for genetic and phenotypic variance explained by each top SNP indicated that more than 50% of the resistance trait is controlled by the QTL on chromosome 21 (Table 2). Distribution of three different genotypes in moribund and surviving groups is shown in Figure 4.

**TABLE 2:** The top SNPs at chromosome 21 of rainbow trout, showing significant association to higher resistance against *V. anguillarum*.

**Chromosome**	**SNP**	**Position**	**Type**	**Freq.**	**Effect**	**SE**	**P**	**Fr. genvar.**	**Fr. phenvar.**
21	AX-89945921	14114149	G/A	0.57	−0.29	0.03	5.77e–22	77%	17%
21	AX-89918956	15109525	C/A	0.51	−0.25	0.03	2.52e–18	57%	13%
21	AX-89969941	13976778	C/T	0.46	−0.26	0.03	1.92e–17	61%	14%
21	AX-89934035	14608563	C/T	0.35	−0.26	0.03	6.18e–16	56%	12%

*The two last columns give the estimated fraction of genetic and phenotypic variance explained by each SNP.*

### Gene Expression

#### Gills

**FIGURE 5:**
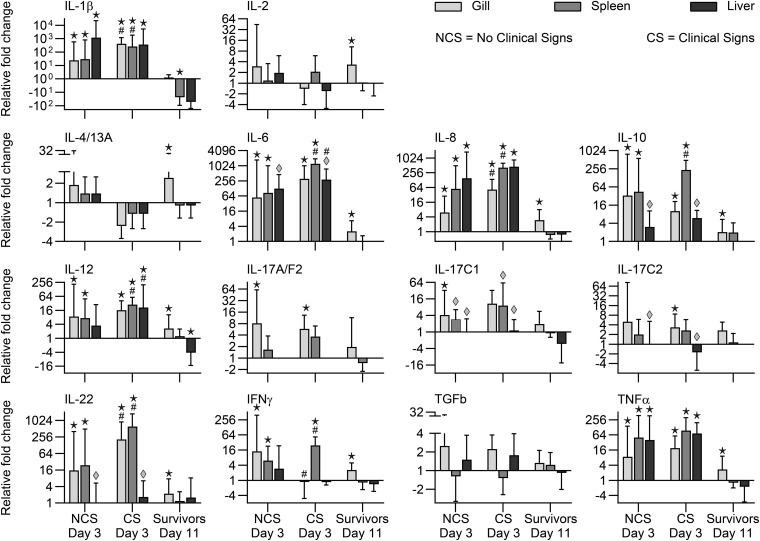
Expression of cytokine genes in rainbow trout exposed to *V. anguillarum*. NCS, no clinical signs; CS, clinical signs; Survivors, Fish surviving exposure to the pathogen 11 dpe. ★ : Significant differences (*p* < 0.01) between exposed and non-exposed (control) groups (NCS, CS, and surviving groups shown). ^#^Significant differences (*p* < 0.01) between the NCS and CS groups at 3 dpe. ◆:Significant differences (*p* < 0.01, non-parametric Mann–Whitney test) between exposed and control groups.

**FIGURE 6:**
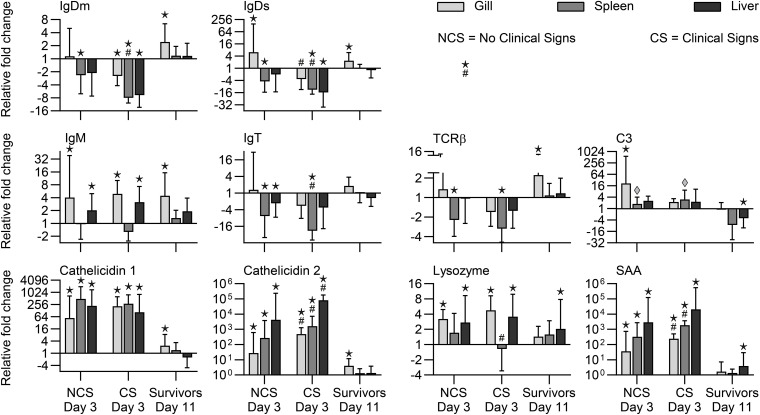
Expression of effector molecule genes in rainbow trout exposed to *V. anguillarum*. NCS, no clinical signs; CS, clinical signs; Survivors, Fish surviving exposure to the pathogen 11 dpe. ★ : Significant differences (*p* < 0.01) between exposed and non-exposed (control) groups (NCS, CS, and surviving groups shown). ^#^Significant differences (*p* < 0.01) between the NCS and CS groups at 3 dpe. ◆:Significant differences (*p* < 0.01, non-parametric Mann–Whitney test) between exposed and control groups.

When comparing expression of the genes between NCS and CS fish (3 dpe), we recorded significantly higher transcript levels in CS fish of genes encoding IL-1β, IL-6, IL-8, IL-22, Cath1, Cath2, and SAA. Surviving fish (11 dpe) showed higher expression in gills of genes encoding IgDm, IgDs, and TCR together with genes encoding cytokines (IL-2, IL-4/13A, IL-6, IL-8, IL-10, IL-12, IL-17C2, IL-22, TNF) and effector molecules IgM, Cath-1, and Cath-2 (Figures 5, 6).

#### Spleen

Significant higher expression of cytokine genes for IL-1β, IL-6, IL-8, IL-10, IL-12, IL-22, TNF, and effector molecules C3, Cath1, Cath2, SAA were observed in spleen samples in both NCS and CS fish with the highest folds for SAA, Cath2 and IL-6 (1876-fold, 1640-fold, 1234-fold, respectively) in CS fish. Genes encoding cytokines (IL-1β, IL-8, IL-10, IL-12, IL-17C1, IL-22, IFNγ) showed significantly higher level in CS fish compared to NCS fish. Genes encoding IgDm, IgDs, IgM, IgT, TCR, and TGFβ showed lower level in NCS and CS fish (Figures 5, 6).

#### Liver

Both fish groups (NCS and CS) showed higher level of Cath-2 and SAA genes in this organ. Cytokine genes encoding IL-1β, IL-6, IL-8, IL-10, IL-12, IL-17C1, IL-17C2, IL-22, TNFα and the gene encoding IgM were also showing higher level in both CS and NCS fish livers. IgDm, IgDs and IgT genes showed lower level in the liver of both groups. Lysozyme and SAA genes remained high in surviving fish (Figures 5, 6).

#### Bacterial Load

**FIGURE 7:**
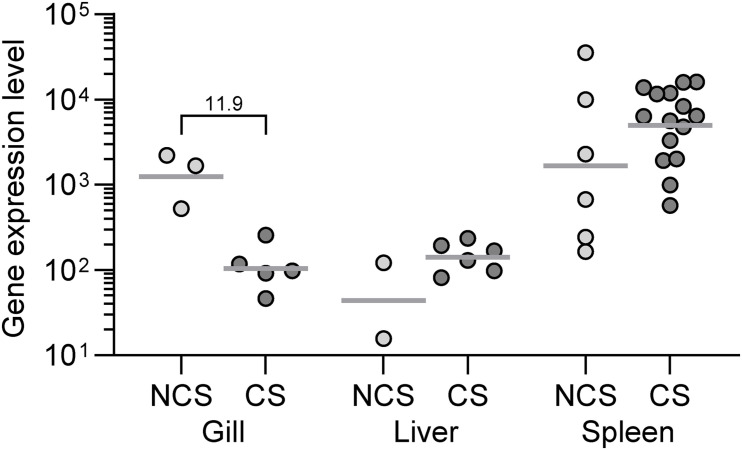
The level of bacterial (*V. anguillarum*) recA transcripts calculated as 2^– Δ^ ^Cq^ in rainbow trout exposed to the live bacterium. Fish showing clinical signs (CS) at 3 dpe compared to fish showing no clinical signs (NCS) at the same time point. Surviving fish at 11 dpe did not show transcripts of the *V. anguillarum* recA gene. Numbers above the brackets indicate the fold difference between the CS and NCS groups.

The infection levels in different organs of the fish at different time points are depicted as the level of transcription of the bacterial gene *recA* (Figure 7). We found generally a higher number of bacterial transcripts in the spleen compared to liver and fish with clinical signs showed a higher transcript level compared to fish without disease signs. In gills the bacterial load reflected as *recA* expression was higher in NCS than CS. No bacterial transcripts were recorded in any organ of the surviving fish.

An overview of the gene expression from all genes in all samples are presented in the Supplementary Table 2.

### Location of Genes in the Trout Genome

To evaluate the candidate genes (both genes studied by qPCR and others) involved in *V. anguillarum* resistance located on Omy 21, the annotated protein coding genes in RefSeq (Accession GCA_002163495.1) were investigated with focus on the discovered QTL region (13,600,00–14,600,00 bp). Results are shown in the Supplementary Figure 1. Among 35 protein-coding genes, one pseudogene and 2 non-coding RNA genes were identified in the region (Supplementary Table 3), and the gene encoding protein phosphatase 1 regulatory subunit 12A contains the most significant SNP (AX-89945921). A particularly relevant finding was the presence of different variables of genes encoding immunoglobulin (Ig) light chain kappa in close association with the significant SNP.

## Discussion

In the present work, the genetic analyses of susceptible versus resistant fish showed the potential of genetic selection of resistant fish as a method to improve natural disease resistance in rainbow trout. The mortality flattened out before termination of the challenge period and left more than 40% survivors, which is optimal for the subsequent statistical analysis. In addition, the moderate mortality maximizes phenotypic variation whereby genetic differences in resistance become more evident. The daily mortality approaching zero toward the end of the test indicates that survivors are truly resistant, not merely fish with longer incubation period, or fish that otherwise delay mortality without being able to survive in the long run. We found that the major QTL associated with resistance toward *V. anguillarum* were clustered on rainbow trout chromosome 21 (Omy 21). It differs from QTL associated with *F. psychrophilum* resistance on chromosome 19 (Omy19) (Wiens et al., 2013) and toward the parasitic ciliate *Ichthyophthirius multifiliis* on chromosome 16 and 17 (Omy 16 and 17) (Jaafar et al., 2020) confirming that resistance mechanisms in a specific fish species toward different pathogens differ considerably (Fraslin et al., 2020). In the present work, the favorable homozygote (considering the top SNP) had an overall mortality of 37%, while the opposite homozygote had a mortality of 87%. The heterozygote (Qq) had an almost similar resistance (45% mortality) as the favorable homozygote (QQ). In the current study, testing was continued until mortality had naturally ceased, indicating that survivors cleared the pathogen. To evaluate the candidate genes involved in *V. anguillarum* resistance located on Omy 21, the annotated protein coding genes were investigated with focus on the discovered QTL region. Among 35 protein coding genes, one pseudogene and 2 non-coding RNA genes were identified in the region, and the gene encoding protein phosphatase 1 regulatory subunit 12A contains the most significant SNP (AX-89945921). A particularly immune relevant finding was the presence of different segments encoding immunoglobulin (Ig) light chain kappa in close association with the significant SNP - suggesting the potential role of these segments in protection. Thus, in our gene expression study, we showed that trout surviving the *V. anguillarum* exposure exhibited an elevated expression of IgM, IgDm and IgDs in gills. It is noteworthy that immunoglobulin genes recently were suggested to play a role in protection of Atlantic salmon against SAV (Hillestad et al., 2020). As the light chains are part of the antigen binding site, it can be speculated that some of the kappa light chains contribute to a *V. anguillarum* specific site. Support for this notion is found in previous studies which mapped four Ig light chains isotypes (IgL1, IgL2, IgL3, and IgL4) gene segments to be co-localized on chromosomes 21, 18, 15, 7, 13, and 17 of rainbow trout representing a number of constant (C), variable (V) and Joining (J) regions (Rego et al., 2020). Hence, genomic differences in the IgL repertoire between different lines of trout suggested a potential role of these isoforms causing immune response diversity in rainbow trout (Rego et al., 2020). Genetic mapping of the Japanese flounder genome highlighted 12 immune-related genes associated with *V. anguillarum* resistance; These comprised genes encoding tap1, satb1, cd40, and cd69, known as molecular organizers for the major histocompatibility complex (MHC) class I and II (Shao et al., 2015). Analyses of the tongue fish genome indicated that resistance to *V. anguillarum* was associated with MHC II B alleles (Du et al., 2011), whereas Zhang et al. (2019) found that several genes in turbot, connected to inflammation and host membrane protection against bacterial entry (PI3K/Akt/mTOR and NF-kB pathways), were associated with resistance. However, although immune mechanisms are likely to play a major role in disease resistance it should be framed that numerous interactions occur between a certain pathogen and the host. Various cellular proteins and metabolic pathways are involved whereby the interpretation of QTL results is difficult since it identifies polymorphisms on loci not the actual effective mutation (Fraslin et al., 2020).

We measured the *V. anguillarum* infection level in exposed rainbow trout and found that surviving fish were free from infection (all organs). This indicates that the resistant fish have mechanisms to eliminate or at least inactivate the pathogen. The spleen in both susceptible fish (showing clinical signs) and more resistant fish at an early time point (showing no clinical signs) harbored more bacteria in the spleen compared to liver, which illustrates that the spleen is a central immune organ when combatting infections (Zuo et al., 2020). It is noteworthy, that gills from fish with no clinical signs at an early time point showed a higher *V. anguillarum* colonization. This may suggest that local protective mechanisms, including a thick sticky mucus layer, may not only prevent the bacteria from penetrating the gills, but also accumulate surrounding bacteria.

As the rainbow trout exposed to *V. anguillarum* exhibited very different susceptibilities to infection it was important to investigate the genes activated during the course of infection. We measured the expression of immune relevant genes in fish with and without clinical signs, sampled at the same time point post-exposure. It was generally observed that fish with clinical signs showed a higher spleen load of bacteria and a higher expression of immune genes compared to fish without clinical symptoms. This suggests that the pathogen load is correlated to the level of response. Various fish species have been exposed to *V. anguillarum* experimentally, and it is generally observed that immune genes are regulated following infection. Our gene expression results are in line with data from corresponding studies on Atlantic cod (Caipang et al., 2008; Seppola et al., 2008), seabream (López-Castejón et al., 2007) and sea bass (Meloni et al., 2015). The first reaction to bacterial exposure is an induction of inflammation involving expression of pro-inflammatory cytokines. Subsequently, other humoral processes are initiated, among others, upregulation of genes encoding IgM in rainbow trout (Khansari et al., 2019) and complement in yellow croaker (Wei et al., 2009), respectively. Clear differences were found between internal organs and gills but in general, the immune gene expression profile included a high expression of inflammatory cytokine genes as commonly seen during many bacterial and parasitic infections (Zuo et al., 2020). In addition, genes encoding effector molecules (immunoglobulins, antimicrobial peptides, cathelicidins, acute phase reactants such as SAA and lysozyme) were expressed at a higher level. These genes may of course not be directly or primarily involved but may provide a clue to reaction patterns in resistant fish. It was therefore noteworthy that mainly gills in the surviving fish showed a higher expression level of cytokine genes encoding IL-2, IL-4/13A, IL-6, IL-8, IL-10, TNF-α, IFNγ and effector molecule genes encoding IgDm, IgDs, IgM, Cathelicidins I and II, and TCRβ. This illustrates that the immune reactions in gills are decisive elements in the host response to waterborne pathogens. Both humoral and cellular factors may take part in the response and this involves both innate immune cells (e.g., neutrophils) and antigen presenting cells (Kato et al., 2018) but also lymphocytes involved in production of immunoglobulins. Several of the cytokine genes (encoding IL-6, IL-8, IL-10, TNF-α, and IFNγ) activated in gills of the survivors are central in the inflammatory reactions and are often induced by the pathogen (Zou and Secombes, 2016). Others play a more direct role in adaptive immunity. IL-2 is considered a T cell growth factor but it has more intricate effects on T cell subsets and thereby both antibody responses and Natural Killer cell activity (Oppenheim, 2007). The cytokine also regulates the expression of the IL-4/13A gene which is a central regulator of antibody production (Zou and Secombes, 2016). It is well known that IgM is an important effector molecule in fish (Thornburn and Jansson, 1988; Holten-Andersen et al., 2012) but the consistent activation of genes encoding IgD (membrane bound and secreted form) is particularly important. This IgD immunoglobulin class has been known for decades but its function is not adequately defined. The IgD gene was not activated in trout gills exposed to another bacterial pathogen *Yersinia ruckeri* (Zuo et al., 2020) suggesting a relatively specific association of this gene with *V. anguillarum* clearance from the gill surface. Another immunoglobulin class (IgT), which is considered to play a major role in mucosal surfaces (Xu et al., 2013; Zhang et al., 2017; Yu et al., 2018), was found expressed at a lower level in all samples from CS fish. This suggests that the infection strategy of *V. anguillarum* differs from other pathogens and it cannot be excluded that IgD rather than IgT is involved in protection of the fish surface against *V. anguillarum*. The gene encoding the T-cell receptor TCRβ was also expressed at a higher level, which complies with the abundant presence of T-lymphocytes in salmonid gills (Koppang et al., 2010). This suggests the importance of adaptive mechanisms in the natural resistance to *V. anguillarum* in rainbow trout. The involvement of genes encoding antimicrobial peptide genes such as cathelicidins (Furlan et al., 2018), underline the importance of these innate pathogen limiting peptides. Some of them may have direct effects on pathogens elimination (Munoz-Atienza et al., 2019), and were found to be involved in the response of trout toward *Y. ruckeri* (Zuo et al., 2020).

It is relevant to compare the results from the rainbow trout SNP analyses with the gene expression profiles of surviving fish in order to find indications of immune gene involvement in *V. anguillarum* resistance. The above mentioned results show that genes encoding immunoglobulins, antimicrobial peptides and various T-cell types were regulated in surviving fish. However, in the cross-field between genome and expression analyses the immunoglobulins were indicated to be highly involved in natural resistance of this fish host.

## Conclusion

The present study has pointed the presence of a significant QTL, localized on chromosome 21 (Omy21) which is involved in natural resistance of rainbow trout against *V. anguillarum*. This QTL provides a basis for selection of spawners (females and males) for future production of relatively resistant fish for aquaculture. The genetic analysis indicated the heterozygous (Qq) fish to be almost as resistant as the homozygous (QQ) which allows breeding companies to merely apply QQ sires for breeding. These males can secure a high resistance of offspring despite being heterozygous. Gene expression analyses suggested a number of immune genes to play a role in the protection of fish. Of particular interest are genes encoding immunoglobulins (IgD and IgM), cathelicidins, T-cell receptor equipped cells and the light chain kappa genes associated with the most important SNP.

## Data Availability Statement

The data presented in the study are deposited in the Gene Expression Omnibus (GEO) Database (Accession: GSE158411) and European Variation Archive (EVA) (Accession: Project: PRJEB41769; Analyses: ERZ1689028) repository.

## Ethics Statement

The animal study was reviewed and approved by Experimental Animal Inspectorate, Committee for Experimental Animals, Ministry of Environment and Food, Denmark – License No: 2019-15-0201-01614.

## Author Contributions

KB and TN designed and organized the investigation. ID provided the pathogen. AK, MM, SZ, RJ, HM, KB, and LG followed the exposed fish and sampled. JØ analyzed QTL data. AK, MM, SZ, and HM performed the gene expression studies. PK analyzed qPCR data. AK and KB wrote the manuscript. All authors contributed to the article and approved the submitted version.

## Conflict of Interest

JØ and TN were employed by companies AquaGen and Aquasearch Ova ApS, respectively. The remaining authors declare that the research was conducted in the absence of any commercial or financial relationships that could be construed as a potential conflict of interest.

## Supplementary Material

Supplementary Figure 1 An overview of chromosome 21 (Omyk_1.0) position 13,600,000 to 14,600,000. The two central parallel black lines represent 1 million nucleotides of chromosome 21 at the proximity of major the QTL, AX-89945921 (yellow oval). This QTL is located in the second intron of LOC110500010 which encodes for protein phosphatase 1 regulatory subunit 12A. The blue arrows indicate the position and reading direction of the genes present with the id of loci and protein products; ncRNA indicates non-coding RNA. In green, examples of mRNA are indicated above and under the genes; non-vertical lines indicate introns. Specifically, red indicate 16 mRNAs encoding for immunoglobulin (Ig) kappa V segments within the loci range LOC110500015 to −31 (the red numbers represent the two last digits of the loci number). It is not possible in this presentation to distinguish LOC110500024 and LOC110500031 due to their close proximity and the scaling used.

Supplementary Figure 2 QQ plot.

Supplementary Table 1 List of primers and probes used for qPCR assays investigating gene expression in rainbow trout challenging with *V. angularium*. All nucleotides are from 5′ end (labeled with FAM) to 3′ end (labeled with BHQ1). All the qPCR assays were optimized to have annealing temperature of 60°C and having efficiencies of 100 ± 5%. ^R^indicates reference genes (housekeepers). ^MS^indicates the qPCR assay targets both membrane bound and secreted forms. ^1^The α chain of IL-12 is common to the two isoforms of IL-12. The reference indicate the source of development of the assay. Genes locations were identified on the reference genome (*Oncorhynchus mykiss* Omyk_1.0 - GCA_002163495.1) using NCBI Genom Data Viewer.

Supplementary Table 2 Gene expressions analysis resume.

Supplementary Table 3 List of annotated genes on QTL region, rainbow trout chromosome 21.
