# Supplementary figures and images for: A Major QTL for Resistance to Vibrio anguillarum in Rainbow Trout
Source: Front Genet. 2020 Dec 29;11:607558. doi: 10.3389/fgene.2020.607558 (PMC7802751; doi:10.3389/fgene.2020.607558)

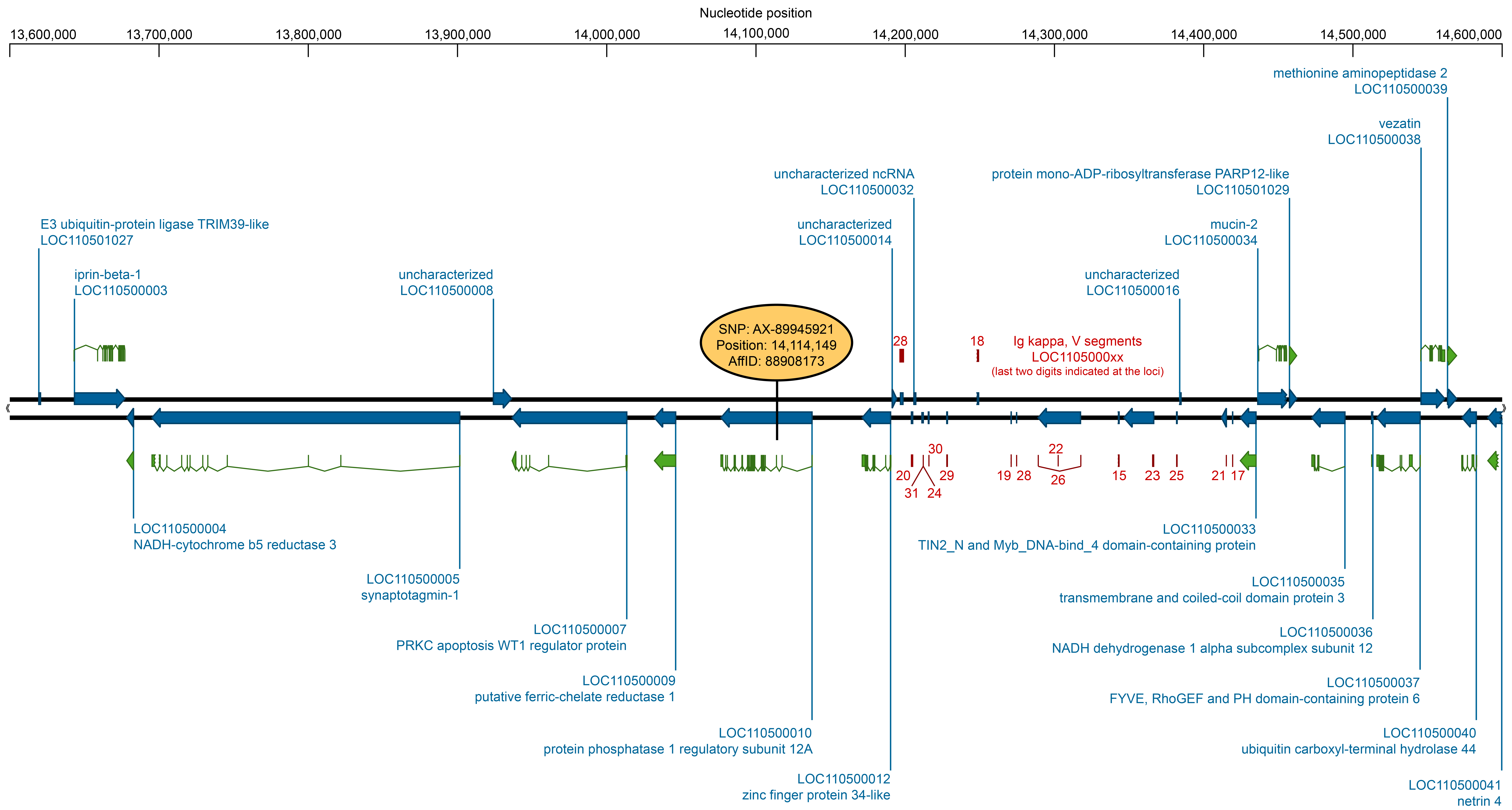

Supplement: Supplementary Figure 1 — An overview of chromosome 21 (Omyk_1.0) position 13,600,000 to 14,600,000. The two central parallel black lines represent 1 million nucleotides of chromosome 21 at the proximity of major the QTL, AX-89945921 (yellow oval). This QTL is located in the second intron of LOC110500010 which encodes for protein phosphatase 1 regulatory subunit 12A. The blue arrows indicate the position and reading direction of the genes present with the id of loci and protein products; ncRNA indicates non-coding RNA. In green, examples of mRNA are indicated above and under the genes; non-vertical lines indicate introns. Specifically, red indicate 16 mRNAs encoding for immunoglobulin (Ig) kappa V segments within the loci range LOC110500015 to −31 (the red numbers represent the two last digits of the loci number). It is not possible in this presentation to distinguish LOC110500024 and LOC110500031 due to their close proximity and the scaling used. [file Image_1.TIF]

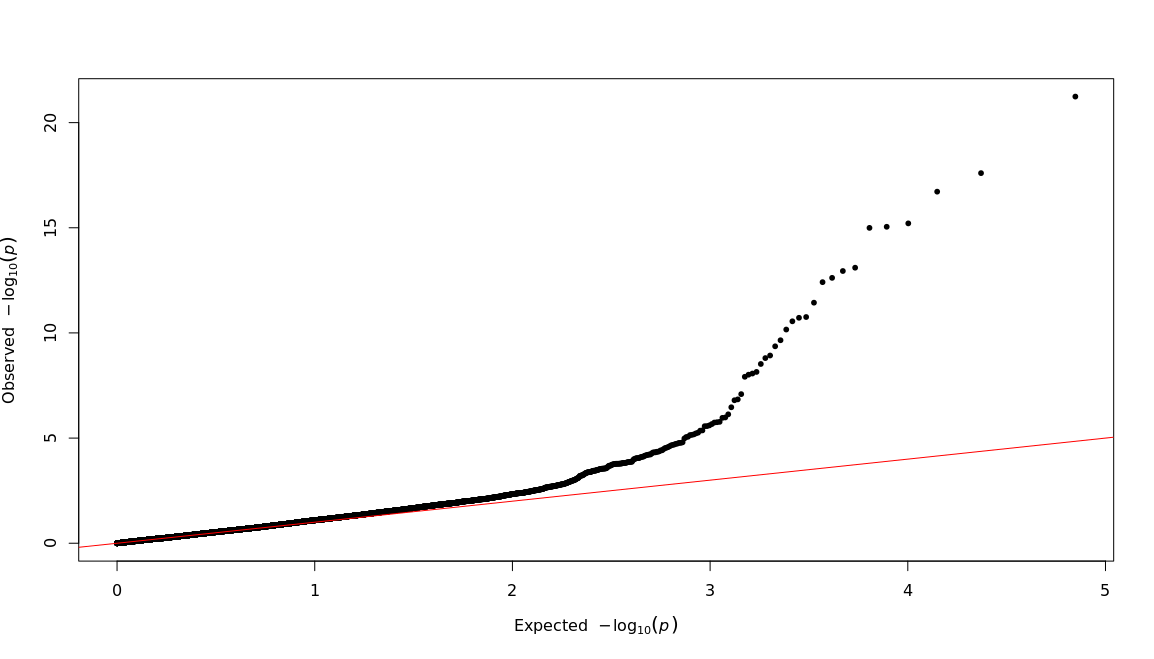

Supplement: Supplementary Figure 2 — QQ plot. [file Image_2.PNG]
