# Supplementary material for: A Major QTL for Resistance to Vibrio anguillarum in Rainbow Trout
Source: Front Genet. 2020 Dec 29;11:607558. doi: 10.3389/fgene.2020.607558 (PMC7802751; doi:10.3389/fgene.2020.607558)
Supplement: Supplementary Table 1 — List of primers and probes used for qPCR assays investigating gene expression in rainbow trout challenging with V. angularium. All nucleotides are from 5′ end (labeled with FAM) to 3′ end (labeled with BHQ1). All the qPCR assays were optimized to have annealing temperature of 60°C and having efficiencies of 100 ± 5%. Rindicates reference genes (housekeepers). MSindicates the qPCR assay targets both membrane bound and secreted forms. 1The α chain of IL-12 is common to the two isoforms of IL-12. The reference indicate the source of development of the assay. Genes locations were identified on the reference genome (Oncorhynchus mykiss Omyk_1.0 - GCA_002163495.1) using NCBI Genom Data Viewer. [file Table_1.DOCX]

**Supplementary Table S1**

List of primers and probes used for qPCR assays investigating gene expression in rainbow trout challenging with *V. angularium*. All nucleotides are from 5’ end (labeled with FAM) to 3’ end (labeled with BHQ1). All the qPCR assays were optimized to have annealing temperature of 60°C and having efficiencies of 100% ± 5%. ^R^ indicates reference genes (housekeepers). ^MS^ indicates the qPCR assay targets both membrane bound and secreted forms. ^1^ The α chain of IL-12 is common to the two isoforms of IL-12. The reference indicate the source of development of the assay. Genes locations were identified on the reference genome (*Oncorhynchus mykiss* Omyk_1.0 - GCA_002163495.1) using NCBI Genom Data Viewer.

| **Gene**  **GenBank acc.no.** | **Length**  **Bp** | **Primers**  **5’end to 3’end** | **Probes**  **5’end to 3’end** | **References** | **Genomic localisation** | |
| --- | --- | --- | --- | --- | --- | --- |
|  |  |  |  |  | **Chromosome & position of amplicon** | **GenBank acc.no.** |
| ^R^ ARP  AY505012 | 106 | Fwd: GAAAATCATCCAATTGCTGGATG  Rev: CTTCCCACGCAAGGACAGA | CTATCCCAAATGTTTCATTGTCGGCGC | Purcell et al., 2004 | chr17: 40340265...40340360 | NC_035093 |
| ^R^ β-actin  AB196465 | 241 | Fwd: ACATCAAGGAGAAGCTGTGCTAC  Rev: TACGGATGTCCACGTCACAC | CCTCTCTGGAGAAGAGCTACGAGCTG | Sugiura et al., 2007; Marana et al., 2017 | chr1: 8515…8865 | NC_035077 |
| ^R^ ELF-1α  [AF498320](http://www.ncbi.nlm.nih.gov/entrez/viewer.fcgi?db=nucleotide&val=20269865) | 63 | Fwd: ACCCTCCTCTTGGTCGTTTC  Rev: TGATGACACCAACAGCAACA | GCTGTGCGTGACATGAGGCA | Ingerslev et al., 2006 | chr14: 30894810...30894861 | NC_035090 |
| C3.3 & C3.4  AF271080 / U61753 | 85 | Fwd: ATTGGCCTGTCCAAAACACA  Rev: AGCTTCAGATCAAGGAAGAAGTTC | TGGAATCTGTGTGTCTGAACCCC | Raida & Buchmann, 2009 | chr17: 18017732...18017796 chr13: 22193859...22193923 | NC_035093  NC_035089 |
| Cathelicidin 1A  AY382478 | 189 | Fwd: TCTCTCGTCCTGGGGTT  Rev: GTTGTAGCGTGCTGATCTATG | TAATTGGTCGTCCTGGGGGTGG | Marana et al., 2017 | chr28: 12847647...12847835 | NC_035104 |
| Cathelicidin 2A  AY360356 | 135 | Fwd: AAAGATTCCAAGGGGGGT  Rev: CAAAGGGTGTGTTGTGCTGT | GCTCTCGTCCTGGGTTTGGCTCC | Xueqin et al., 2012 | chr8:67206588...67206722 | NC_035084 |
| IFN γ1 and IFN γ2  FJ184374 / FJ184375 | 68 | Fwd: AAGGGCTGTGATGTGTTTCTG  Rev: TGTACTGAGCGGCATTACTCC | TTGATGGGCTGGATGACTTTAGGA | Raida & Buchmann, 2007 | unplaced genomic scaffold: 1160...1227 | NW_018573689 |
| IgDm  AY870262 | 304 | Fwd: CAGGAGGAAAGTTCGGCATCA  Rev: CCTCAAGGAGCTCTGGTTTGGA | CCACACCACACAGACTCTGGCCCTGAA | Skov et al., 2018 | chr13: 48059335...48383504 | NC_035089 |
| IgDs  JQ003979 | 304 | Fwd: TGGCACGCCAGGATTTGAC  Rev: TCAGAATTGAGTGAACGGACAGACA | CCACACCACACAGACTCTGGCCCTGAA | Skov et al., 2018 | chr13: 48059371...48059486 | NC_035089 |
| ^MS^ IgM  S63348 / AH014877 | 72 | Fwd: ACCCTCCTCTTGGTCGTTTC  Rev: TGATGACACCAACAGCAACA | TGATGACACCAACAGCAACA | Raida & Buchmann, 2007 | chr12: 81780295...81780366 | NC_035088 |
| ^MS^ IgT  AY870265 / AY870263 | 73 | Fwd: AGCACCAGGGTGAAACCA  Rev: GCGGTGGGTTCAGAGTCA | AGCAAGACGACCTCCAAAACAGAAC | Raida & Buchmann, 2007 | chr13: 48285298...48285493 | NC_035089 |
| IL-1β  AJ223954 | 91 | Fwd: ACATTGCCAACCTCATCATCG  Rev: TTGAGCAGGTCCTTGTCCTTG | CATGGAGAGGTTAAAGGGTGGC | Raida & Buchmann, 2007 | chr6: 42311100...42311190 | NC_035082 |
| IL-2a  FJ571513 | 110 | Fwd: ATGCAACACCACATCAGCAT  Rev: TGCCACGGCCCTACAAAAGA  RE  TGCCACGGCCCTACAAAAGA | TGCCACGGCCCTACAAAAGA | Marana et al., 2017 | chr25: 30109976...30110224 | NC_035101 |
| IL-4/13a  AB574337 | 138 | Fwd: ATCCTTCTCCTCTCTGTTGC  Rev: GAGTGTGTGTGTATTGTCCTG | CGCACCGGCAGCATAGAAGT | Chettri et al., 2014 | chr12: 50133310...50133448 | NC_035088 |
| IL-6a  DQ866150 | 91 | Fwd: ACTCCCCTCTGTCACACACC  Rev: GGCAGACAGGTCCTCCACTA | CCACTGTGCTGATAGGGCTGG | Raida & Buchmann, 2008 | chr14: 7102251...7102305 | NC_035090 |
| IL-8 isoforms a, b , c, d & e  AY160982 to AY160986 | 69 | Fwd: AGAATGTCAGCCAGCCTTGT  Rev: TCTCAGACTCATCCCCTCAGT | TTGTGCTCCTGGCCCTCCTGA | Raida & Buchmann, 2008 | chr12: 74891818...74891878 chr12: 74911280...74911340 chr: 13: 41793683...41793743 | NC_035088 NC_035088 NC_035089 |
| IL-10a  [AB118099](http://www.ncbi.nlm.nih.gov/entrez/viewer.fcgi?db=nucleotide&val=47678892) | 70 | Fwd: CGACTTTAAATCTCCCATCGAC  Rev: GCATTGGACGATCTCTTTCTTC | CATCGGAAACATCTTCCACGAGCT | Raida Raida & Buchmann, 2007 | chr12: 50133310...50133448 | NC_035088 |
| ^1^ IL-12 α chain  HE798148 | 84 | Fwd: CAACGGAACACCACATTCAG  Rev: AGCCTGTAGTGAGGCAGCAT | TGCGTGTCTGAGGAACATCCG | Jaafar et al., 2015 | chr14: 7102251...7102305 | NC_035090 |
| IL-17A/F2a  AJ580842 | 158 | Fwd: TCAAAAGCAACGTGTCGAAG  Rev: TCCCTCTGATTCCTCTGTGG | TATGCTGCTGGGCCTGACCA | Jaafar et al., 2015 | chr19: 32909499...32910190 | NC_035095 |
| IL-17c1  CAW30792 | 138 | Fwd: CTGGCGGTACAGCATCGATA  Rev: GAGTTATATCCATAATCTTCGTATTCGGC | CGTGATGTCCGTGCCCTTTGACGATG | Chettri et al., 2014 | chr1: 22090819...22090948 | NC_035077 |
| IL-17c2  CAW30793 | 134 | Fwd: CTGGCGGTACAGCATCGATA  Rev: CAGAGTTATATGCATGATGTTGGGC | CGTGGTGTCCAGGCCCTTTAATGATG | Chettri et al., 2014 | chr2: 51653897...51654020 | NC_035078 |
| IL-22  AM748537 | 64 | Fwd: ATGACCACCACCACAGCATT  Rev: ATTCCTTTCCCCTCCTCCAT | CTTTCCGCAAGAAGTTGTCCGAG | Olsen et al., 2011 | chr13: 50267165...50267390 | NC_035089 |
| Lysozyme CII  X59491 | 188 | Fwd: GAAACAGCCTGCCCAACT  Rev: GTCCAACACCACACGCTT | ATACCCAGGCCACCAACCGCAACAC | Chettri et al., 2012 | chr5: 6920839...6922770 chr5: 6935710...6937091 | NC_035081  NC_035081 |
| SAA  AM422446 | 79 | Fwd: GGGAGATGATTCAGGGTTCCA  Rev: TTACGTCCCCAGTGGTTAGC | TCGAGGACACGAGGACTCAGCA | Skov et al., 2012 | chr6: 67564044...67564107 | NC_035082 |
| TCR-β  AF329700 | 73 | Fwd: TCACCAGCAGACTGAGAGTCC  Rev: AAGCTGACAATGCAGGTGAATC | CCAATGAATGGCACAAACCAGAGAA | Raida Raida & Buchmann, 2007 | chr6: 67564044...67564107 | NC_035082 |
| TGF-β1a  [X99303](http://www.ncbi.nlm.nih.gov/entrez/viewer.fcgi?db=nucleotide&val=1478246) | 75 | Fwd: TCTGAATGAGTGGCTGCAAG  Rev: GGTTTCCCACAATCACAAGG | CTGGAGAGGAGCAGGGATTCCAAT | Raida Raida & Buchmann, 2007 | chr29: 30680564...30681150 | NC_035105 |
| TNF-α1 & TNF-α2  AJ277604 / AJ401377 | 75 | Fwd: GGGGACAAACTGTGGACTGA  Rev: GAAGTTCTTGCCCTGCTCTG | GACCAATCGACTGACCGACGTGGA | Raida & Buchmann, 2008 | chr3: 29836438...29836503 chr2: 19874096...19874161 | NC_035079 NC_035078 |
| recA (*V. angularium*))  LC370212 | 248 | Fwd: ATCGCGGCTCCCTTTAAACA  Rev: AGAGAATCCAGCCGCCGCCATGG | AACTCGGCTGGATTGAGCAG | This study | Not relevant | Not relevant |
